# Harnessing photosynthesis to produce electricity using cyanobacteria, green algae, seaweeds and plants

**DOI:** 10.3389/fpls.2022.955843

**Published:** 2022-07-27

**Authors:** Yaniv Shlosberg, Gadi Schuster, Noam Adir

**Affiliations:** ^1^Grand Technion Energy Program, Technion - Israel Institute of Technology, Haifa, Israel; ^2^Schulich Faculty of Chemistry, Technion - Israel Institute of Technology, Haifa, Israel; ^3^Faculty of Biology, Technion - Israel Institute of Technology, Haifa, Israel

**Keywords:** photosynthesis, clean energy, bio-photogenerator, seaweeds, green algae, cyanobacteria, electricity, bio-photoelectric cell

## Abstract

The conversion of solar energy into electrical current by photosynthetic organisms has the potential to produce clean energy. Life on earth depends on photosynthesis, the major mechanism for biological conversion of light energy into chemical energy. Indeed, billions of years of evolution and adaptation to extreme environmental habitats have resulted in highly efficient light-harvesting and photochemical systems in the photosynthetic organisms that can be found in almost every ecological habitat of our world. In harnessing photosynthesis to produce green energy, the native photosynthetic system is interfaced with electrodes and electron mediators to yield bio-photoelectrochemical cells (BPECs) that transform light energy into electrical power. BPECs utilizing plants, seaweeds, unicellular photosynthetic microorganisms, thylakoid membranes or purified complexes, have been studied in attempts to construct efficient and non-polluting BPECs to produce electricity or hydrogen for use as green energy. The high efficiency of photosynthetic light-harvesting and energy production in the mostly unpolluting processes that make use of water and CO_2_ and produce oxygen beckons us to develop this approach. On the other hand, the need to use physiological conditions, the sensitivity to photoinhibition as well as other abiotic stresses, and the requirement to extract electrons from the system are challenging. In this review, we describe the principles and methods of the different kinds of BPECs that use natural photosynthesis, with an emphasis on BPECs containing living oxygenic photosynthetic organisms. We start with a brief summary of BPECs that use purified photosynthetic complexes. This strategy has produced high-efficiency BPECs. However, the lifetimes of operation of these BPECs are limited, and the preparation is laborious and expensive. We then describe the use of thylakoid membranes in BPECs which requires less effort and usually produces high currents but still suffers from the lack of ability to self-repair damage caused by photoinhibition. This obstacle of the utilization of photosynthetic systems can be significantly reduced by using intact living organisms in the BPEC. We thus describe here progress in developing BPECs that make use of cyanobacteria, green algae, seaweeds and higher plants. Finally, we discuss the future challenges of producing high and longtime operating BPECs for practical use.

## Introduction

There is an increasing concern about the adversities that may occur due to global climate change. To fight this phenomenon, extensive actions are being taken to replace polluting energy production technologies with cleaner ones. One of the dominant environmental factors that are considered a risk are carbon emissions to the atmosphere by energy technologies that involve a combustion process. For this reason, an enormous scientific effort is being conducted to invent new energy technologies that do not involve combustion. Among these technologies are air turbines (Lenzen and Munksgaard, 2002), hydraulic turbines (Guo et al., 2019), solar cells (Wu et al., 2021), fuel cells (Lucia, 2014) and nuclear power plants. These approaches are already in wide usage around the globe.

An interesting approach is the utilization of biomaterials as a source of energy. This could be achieved by isolation of energy-producing organelles such as mitochondria and using them as electron donors in bio-electrochemical cells (Arechederra and Minteer, 2008). Furthermore, certain enzymes such as hydrogenases and nitrogenases can be used to produce hydrogen gas that can be stored and used for energy production in hydrogen fuel cells (Herkendell et al., 2017; Redding et al., 2022). Approaches based on these concepts suggest the utilization of wastewater that naturally contains enzymes and metabolites that are capable of charge transfer (Herkendell, 2021).

**Figure 1:**
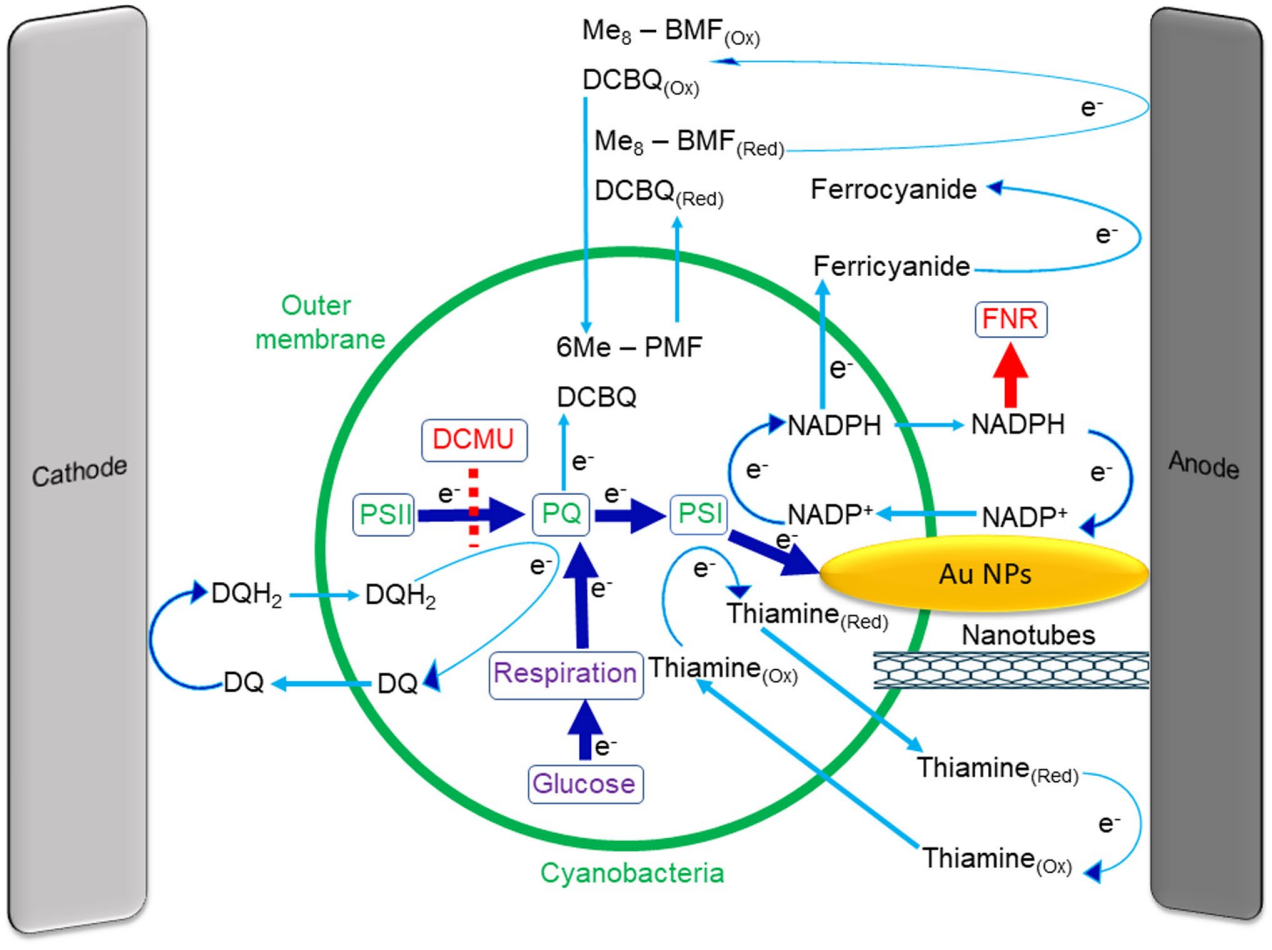
Internal and external mediators that are used in cyanobacterial BPECs. In the photosynthetic pathway, electrons are transferred from water *via* PSII to the plastoquinone pool (PQ) and this is inhibited by the herbicide DCMU (dashed red line). The PQ pool also accepts electrons from the respiratory pathway which is enhanced by the addition of glucose to the cells. The PQH_2_ donates electrons to PSI that photo-reduces NADP^+^ to NADPH. The NADPH molecules can exit the cyanobacteria and reduce the external anode. Then it re-enters the cell to accept additional electrons from PSI. The addition of the NADPH binding protein ferredoxin NADP reductase (FNR) to the external medium inhibits the current by tightly binding the NADPH molecules outside the cell and thereby eliminating the electrical current production (red arrow). The photocurrent generation can be enhanced by the addition of exogenous NADP^+^ or Thiamine, both cycle electrons between PSI and the external anode. The polymer Me_8_–PFM and the soluble quinone DCBQ can enter the cells and cycle electrons between the PQ pool and the external anode. The artificial added mediator Fe(CN)_6_ significantly enhances the current. However, it does not enter the cells and accepts electrons from NADPH at the external surface of the cells. Direct electron transfer is achieved by a direct linkage between the inner part of the cells and the external anode, for example by using nanotubes. Rather than using electron mediators to extract electrons from the cells to the anode, it is also possible to internalize electrons into the photosynthetic pathway. This can be done by reduction of DQ to DQH_2_ by the cathode, while DQH_2_ can enter the cells, donate electrons to the PQ pool, and re-exit the cell to accept additional electrons from the cathode. A green circle represents the cyanobacteria. Blue arrows represent the direction of the electron transport between components.

Rather than using metabolites and proteins, whole bacterial cells can also be applied as an energy source in microbial fuel cells. This promising approach was first implemented by integrating bacteria with electrochemical cells (Ieropoulos et al., 2005). Bacterial cells can perform external electron transport to reduce the anode (Park et al., 1999; Fang et al., 2020) or accept electrons from the cathode (Gregory et al., 2004; Bergel et al., 2005; Fang et al., 2020). Electron transfer is performed by direct or mediated electron transfer (DET and MET, respectively; Figure 1). DET is performed by protein complexes that contain series of electron carriers, thylakoids or living cells to the electrode. MET is conducted by intracellular protein complexes that can reduce electroactive metabolites that can exit the cells and reduce the anode to produce an electric current (Hartshorne et al., 2007; Nevin et al., 2008; Yi et al., 2009; Lovley, 2012; Shi et al., 2012; Heidary et al., 2020). The MET current production can be further amplified by the addition of exogenous artificial electron mediators such as thionine, sulfides, cystine, neutral red, ferric chelated complexes, soluble quinones, phenazines, and humic acids (Lovley et al., 1996, 2004; Rabaey et al., 2004, 2005; Ieropoulos et al., 2005; Simoska et al., 2019; Figure 1). Among the bacteria that produce the highest rates of external electron transport (EET) are *Geobacter sulfurreducens* (Nevin et al., 2008; Yi et al., 2009; Lovley, 2012; Heidary et al., 2020) and *Shewanella oneidensis* (Hartshorne et al., 2007; Shi et al., 2012). Microbial fuel cells are not limited to bacteria; they can utilize also other microorganisms, such as yeast (Bahartan et al., 2012).

A further development of the microbial fuel cells technology is bio-photo electrochemical cells (BPEC). In BPECs, photosynthesis is harnessed to convert the light energy to electricity or to produce high-energy chemical compounds. BPECs can utilize isolated photosynthetic components such as thylakoid membranes (Pinhassi et al., 2016), chloroplasts (Hasan et al., 2017), photosystem I (PSI; Gizzie et al., 2015; Caspy et al., 2021; Toporik et al., 2021; Wang et al., 2022), photosystem II (PSII; Sokol et al., 2018; Hartmann et al., 2020; Zhang and Erwin, 2020; Shoyhet et al., 2021) or intact living photosynthetic microorganisms (Shlosberg et al., 2021b; Bombelli et al., 2022; De Moura Torquato and Grattieri, 2022). Unlike non-photosynthetic bacteria, the photosynthetic organisms possess the mechanisms needed to utilize sun light and convert it to electricity (Chen and Blankenship, 2021). In the photosynthetic process, electrons are extracted from water molecules and are transferred through membranal protein-pigment complexes to produce the proton gradient needed for the synthesis of high energy ATP molecules. During photosynthesis, H_2_O and CO_2_ are consumed while O_2_ is produced (Shevela et al., 2019; Blankenship, 2021; Shen, 2021; Yano and Yachandra, 2021; Figure 1). In this review, we discuss the advantages and disadvantages of using different setups and photosynthetic micro and macro-organisms in BPECs. Also, we elaborate on the electron transport mechanisms from the site of photosynthetic process in the cells and organisms to the anode of the electrochemical cell. We discuss the possibility of using exogenous electron mediators or nanoparticles (NPs) to improve the export of electrons from the photosynthetic site. Lastly, we discuss synthetic bio-electrochemical systems that integrate photosynthetic and non-photosynthetic organisms.

## Live microorganisms are more stable in BPECs than isolated photosynthetic components

A major advantage of BPECs that use thylakoid membranes or isolated photosynthetic protein-chlorophyll complexes, such as PSII or PSI, is the absence of physical barriers (membranes or cell walls) that constrain the transport of electron mediators between the photosynthetic component and the anode of the BPEC (Figure 1). Moreover, the thylakoids or photosystems can be tightly attached to the anode (*via* different forms of electrochemically active attachments), forming a pseudo-biofilm that allows the direct and unmediated transfer of electrons to the electrode. In addition, they can be integrated with metal complexes in semi-artificial Z-scheme architectures that convert the absorbed light energy into an electron flow and direct these electrons to the anode (Zhang and Erwin, 2020; Wang et al., 2022). PSII is very sensitive to the photoinhibition of photosynthesis which happens even at low light intensities. While there is a very efficient repair mechanism that rapidly replaces photodamaged PSII in living organisms, it does not operate in thylakoids (Adir et al., 2003; Nishiyama and Murata, 2014; Li et al., 2018). Therefore, BPECs that use thylakoids or PSII complex are efficient in the production of electricity but are short-lived. When using the light intensity needed to saturate the photosynthetic electron flow, the photosynthetic activity dropped in 10 min time scale (Pinhassi et al., 2016; Zhang and Erwin, 2020; Wang et al., 2022). This situation calls for the development of a living organism BPECs where the photoinhibition repair system is fully operational, enabling prolonged (hours or days), operation of the cells.

While non-biological systems can tolerate very harsh conditions such as high temperature, organic solvent solutions, extreme pH, and high light intensities, biological-based electrochemical cells are limited by the environmental and physiological tolerance of the organisms (Zhang et al., 2018; Lewis et al., 2022). A very important technical factor that can enhance the electrical current production is the ionic strength of the electrolyte solution. Increased salinity can increase the conductivity of the BPEC (Shlosberg et al., 2021b). For this reason, there is a significant advantage for BPECs that are based on marine cyanobacteria, among these species is *Acaryochloris marina*. This cyanobacterium is also unique because the photosynthetic complexes contain mostly chlorophyll *d* (as opposed to most cyanobacteria that contain only chlorophyll *a*). This allows it to use near-infrared wavelengths that cannot be used by most cyanobacteria (Loughlin et al., 2013). Nevertheless, the photocurrent production of this species is not much higher than those of freshwater organisms that contain chlorophyll *a* or green microalgae containing chlorophylls *a* and *b* (Shlosberg et al., 2022b). The marine cyanobacterium *Trichodesmium erythraeum*, tolerates high light intensities as it uses air bubbles to float at the surface of the sea where high light intensities are present (Carpenter and Walsby, 1979). These natural properties of this species enable it to produce a photocurrent that is several fold larger than freshwater species (Shlosberg et al., 2022b).

Photocurrent production in BPEC can also be conducted with green micro-algae (Fischer, 2018; Thangam et al., 2021; Shlosberg et al., 2021b; Herrero-Medina et al., 2022). In the eukaryotic photosynthetic microorganisms, the thylakoid membranes are compartmentalized to the chloroplast. Yet, they are able to bypass this barrier (that does not exist in cyanobacteria) and perform light-induced EET. Among the most common microalgal species that have been used in BPECs are *Dunaliella salina* and *Chlorella* sp. (McCormick et al., 2011; Shlosberg et al., 2021b; Herrero-Medina et al., 2022), which are also cultivated in industrial facilities for other purposes like the production of bio-oils, food additives and cosmetics (Yang et al., 2011; Barba et al., 2015; Vieira et al., 2020). Photosynthetic microorganisms use only a small percentage of the energy obtained *via* photosynthesis for EET. In the absence of an exogenous electron mediator, they produce a photocurrent density of only a few μA/cm^2^ (McCormick et al., 2011; Shlosberg et al., 2021b), which is magnitudes of order lower than what is achieved using present technologies such as photovoltaic solar cells (Sarker et al., 2014). On the other hand, the generation of electricity does not reduce the viability of the organisms in the BPEC. This may economically compensate for the low current production, enabling the BPEC technology to be integrated into industrial cultivation facilities without making any reduction to the yield of the crops. As described above, a relatively high photocurrent production can be obtained using the economically important algae *D. salina* that can grow at high salinity in the growth medium (Shlosberg et al., 2021b). Other potentially valuable organisms for microalgae based BPEC are *Chlorella ohadii* and *Chlorella sorokiniana*. *Chlorella ohadii* was isolated from the sand crust of the Israeli desert and characterized as the most rapidly growing photosynthetic eukaryote that is resistant to extreme high light intensities in which other microalgae and plants are photobleached (Levin et al., 2021).

## The factors that affect the photocurrent production when using live organisms

In many cases, non-biological photo-electrochemical setups have been extensively characterized and the chemical reactions in the system are well known. Since the BPECs that are based on living organisms contain a myriad of chemical reactions and metabolites that occur within the cells, it is a much more complicated system which cannot be described in full. In addition, as the cells interact with the electrochemical cell, changes can occur (such as the formation of biofilms) that change the composition of the BPEC even further. Therefore, it is not *a priori* known which reactions take place and dominate in each physiological condition. For example, the organisms can secrete into the external cellular medium (ECM) different amounts of metabolites that can function as mediating electron transfer (MET) molecules to the BPEC anodes, generating electricity. Additional molecules may be reduced on the cathode, leading to current enhancement. Indeed, although many studies about the internal biology of photosynthetic organisms have been conducted for many years, not much is known about the release of molecules to the ECM of these organisms. Components in the electrolyte may influence the performance of the BPEC by interacting with the electrodes or changing the conductivity. In addition, the activity of the BPEC may decrease over time by the effect of fouling (Corpuz et al., 2021), in which the electrode’s surface is clogged by molecules that adhere irreversibly. Increasing the salinity of the electrolyte may affect the amount of fouling, typically increasing metabolite solubility and thus preventing fouling, however some proteins may actually precipitate due to high ionic strength conditions. For those MET molecules that the high salt increases solubility, there may be significant enhancement in current production. However, it should be noted that the ability to use an electrolyte with high salinity in live-organism BPEC is limited by the ability of the organism to thrive.

Another major factor that influences photocurrent formation is the application of a potential bias on the anode. This can be done by using the three-electrode mode with the potentiostat set to apply an external voltage between the working electrode (the anode) and the reference electrode (De Rooij, 2003). Optimization of the applied potential may significantly improve the performance of the BPEC.

An additional factor that affects a specific BPEC performance is the electrode material (Herkendell, 2021; Simeon et al., 2022), as different electrodes may significantly change the photocurrent production *via* specific interactions with the MET molecules. In many cases, non-metallic electrodes such as graphite or fluorine tin-oxide (FTO) are used. Such electrodes may be optimal for analytic use, since they do not corrode, and the measured current is thus not increased due to anode derived electrons. However, under certain conditions these anodes are less conductive and/or less compatible with the presence of photosynthetic organisms and therefore significantly lower photocurrent is obtained. Metals such as iron, stainless steel or aluminum are very good anode material (Bombelli et al., 2022; Shlosberg et al., 2022a). In fact, many organisms in nature perform EET to reduce iron to be able to uptake it up into the cells and use it for the cellular processes.

## NADPH is the major native mediator of live photosynthetic organisms in BPECs

In nature, photosynthesis is the major source of energy production for living organisms. The photosynthetic pathway consists of multiple electron transfer reactions that originate in the extraction of electrons by water splitting and ends in the reduction of NADP^+^ molecules to form NADPH (Shevela et al., 2019; Blankenship, 2021; Figure 1). Most of the NADPH is then used by the Calvin–Benson–Bassham cycle to produce ATP and organic molecules. The secretion of low levels of NADPH and NADH from cyanobacteria, microalgae, and seaweeds following illumination has been identified by 2D- fluorescence measurements of the external media (EM) of the organisms (Shlosberg et al., 2021b, 2022a,b). One might hypothesize that secretion of NADPH by cells would lower their fitness and thus would be avoided. However, it is possible that cells secrete NADPH in order to reduce Fe^3+^ to Fe^2+^ to enable its internalization, as previously described in plant’s roots (Bienfait, 1985). An additional possibility could be that the cells release NADPH into the ECM in order to prevent the presence of reducing equivalents in a strong photosynthetic electron flow situation under high light intensity. Since NADPH secretion is limited, physical, genetic or physiological treatments that weaken the cell membrane could increase in the NADPH dependent electric current in the BPEC (Shlosberg et al., 2021a). A physical method for photocurrent enhancement is achieved by a gentle pressure on the cyanobacterial cells using a microfluidizer, or by the application of a mild osmotic shock (Saper et al., 2018). Some mutations also alter the permeability of the cell wall, leading to an increase in the produced current (Wey et al., 2021; Kusama et al., 2022).

A significant enhancement of NADPH release in cyanobacteria and microalgae to the external medium occurs upon association of the living cells with the anode of a BPEC and application of an electrical bias potential (Shlosberg et al., 2021a). The reason for this is not fully elucidated and may derive from the influence of the applied potential bias that can affect the activity of channels in the cell membrane. Looking from an energetic perspective, the reduction of the anode is spontaneous and therefore may drive NADPH molecules that are present at the internal surface of the cells to exit the cell and reduce the anode. A similar mechanism was reported for the reduction of the external mediator potassium ferricyanide [Fe(CN)_6_] by an internal NADPH that can reach the surface of bacterial cells and reduce the ferricyanide that is located on the outer surface of the cell (Gonzalez-Aravena et al., 2018). As described in the following paragraph, in prokaryotes such as cyanobacteria, the respiratory electron transfer pathway intersects with the photosynthetic electron transfer pathway (see below). NADH and FADH_2_ are produced during respiration to reduce the plastoquinone pool that is shared by the two electron transfer processes. The plastoquinol then donates electrons to the cytochrome *b_6_/f* complex and from there *via* plastocyanin or cytochrome *c*_6_ to photosystem I (PSI) that in a light dependent reaction reduces NADP+ to NADPH, a fraction of which could be released from the cell. As described above, NADPH molecules were identified to accumulate in a light dependent process in the ECM of cyanobacteria (Shlosberg et al., 2021a).

## Both the respiration and photosynthetic electron flow systems contribute to the production of NADPH

One of the ways to enhance the photocurrent production in cyanobacteria is the addition of glucose that can enter the cyanobacterial cells providing continual input to the respiratory system (Saper et al., 2018). Nevertheless, one of the benefits of using photoautotrophs instead of non-photosynthetic bacteria derives from its ability to synthesize its own sugar source using only CO_2_, water and light. This is a great economic advantage that lowers the cost of operation when designing applicative bio-generators on the large scale. Furthermore, the addition of high exogenous sugar quantities may become hazardous because it might enable the growth of contaminating pathogenic bacterial species. The addition of the photosystem II (PSII) inhibitor 3-(3,4-dichlorophenyl)-1,1-dimethylurea (DCMU) to microalgae, seaweeds, and plants eliminates the photocurrent production, indicating that PSII activity is essential for EET to occur in these systems and the electron source is the water-splitting activity of PSII. Interestingly, in cyanobacterial species, the source of electrons seems to be dependent on the bias voltage applied to the BPEC. Under the application of low bias potential to the BPEC, DCMU inhibits the photocurrent, indicating that the source of electrons is the water-splitting activity of PSII (McCormick et al., 2015; Jeuken, 2016; Tschörtner et al., 2019; Shlosberg et al., 2021b; Herrero-Medina et al., 2022). This is similar to the situation in green algae, seaweeds, and higher plants which are all eukaryotes in which respiration and photosynthesis are physically separated to the mitochondria and chloroplast organelles. However, when a relatively high bias voltage is applied (>0.5 V), the photocurrent is doubled by the addition of DCMU (Saper et al., 2018; Shlosberg et al., 2021a). In addition, photocurrent production from a PSII deficient mutant of *Synechocystis* have produced the same photocurrent as the wild type, showing that PSII is not involved in the EET in these conditions (Saper et al., 2018). Further evidence that the source of the electrons under these conditions is from the respiratory chain, was shown by photocurrent inhibition by the application of either iodoacetate, a respiratory inhibitor, or the cytochrome *b_6_/f* inhibitor DBMIB (Saper et al., 2018). The respiration electron flow reduces the plastoquinone (PQ) pool that is shared by the respiration and photosynthesis process in cyanobacteria (Figure 1). The electrons then continue to PSI that reduces NADP^+^ to produce NADPH in the light. As described above some of the NADPH is then exported from the living cell and reduces the anode of the BPEC (Figure 1; Shlosberg et al., 2021a). Recently, a similar phenomenon in which DCMU does not inhibit the current has been observed also in the green alga *Chlorella vulgaris* (Herrero-Medina et al., 2022).

## Application of exogenous electron mediators enhances the photocurrents

A major difficulty and limitation of the organism’s BPEC is that the electrons should be transferred from the thylakoids to the anode *via* the periplasmic membrane and cell wall. Enhancement of the photocurrent production can be achieved by the addition of an exogenous artificial electron mediator. The mediator could essentially work in two ways: in the first one the mediator crosses the cell membranes and enters the cell and the chloroplast. It is then reduced by the photosynthetic apparatus, NADPH or another reducer, moves out of the cell and transfers the electrons to the anode. A different scenario sees the exogenous mediator obtaining electrons at the cell outer membrane, without penetrating the cell (Figure 1). Since only a small amount of NADPH or NADH are exported from the cells, the addition of artificial mediators to cyanobacterial or micro-algal containing BPECs resulted in a significant increase in the photocurrent (Tschörtner et al., 2019; Shlosberg et al., 2021a; Pisciotta and Blessing, 2022). Among the promising candidates for applicative photocurrent generation are species that are already being cultivated in industrial facilities for food or cosmetic purposes such as the cyanobacterium *Spirulina* and the microalgae *D. salina*. In such a case, the addition of NADH or NADPH is favored as they are non-toxic and even considered to be good additives to improve human health. Thiamine (vitamin B1) can transfer electrons from photosynthetic microorganisms, enhancing the photocurrent by the same factor as NADPH (Shlosberg et al., 2021b, 2022b). Interestingly, although thiamine is not involved in the native photosynthetic electron transfer pathway, it was suggested that it can function similarly to NADP^+^ and accept electrons from PSI (Shlosberg et al., 2021b). Additional synthetic mediators are the soluble quinone 2,6-Dichloro-1,4-benzoquinone (DCBQ) that can accept electrons from the PQ pool and transfer them to the external anode (Pinhassi et al., 2015; Hasan et al., 2017; Longatte et al., 2017; Wey et al., 2019; Figure 1). However, the attempts using it in live organisms BPEC indicated limited photoelectric currents. A relatively novel synthetic electron mediator that was also used for studying the circadian clock in cyanobacteria is Me_8_-BMF (Nishio et al., 2014; Figure 1). Like DCBQ, its application to BPEC resulted in low photo-currents.

One of the most extensively used artificial electron mediator is Fe(CN)_6_ which is also used in non-photosynthetic microbial fuel cells (Bombelli et al., 2011; Calkins et al., 2013; Cereda et al., 2014; Nguyen and Bruce, 2014; McCormick et al., 2015; Pinhassi et al., 2015, 2016; Sekar et al., 2016; Leister, 2019; Tschörtner et al., 2019; Grattieri et al., 2020; Teodor et al., 2020; Firoozabadi et al., 2021,c). This mediator does not enter the cells and is believed to accept electrons from NADPH at the external surface of the cells. This molecule enhances the NADPH mediated photocurrent in photosynthetic microorganisms by a factor of ~50–100 (Shlosberg et al., 2021b). A negative aspect of this molecule is that it is considered to be toxic to the cells during long time operating of BEPCs (Figure 1). Another interesting exogenous additive that can enhance the photocurrent are gold nanoparticles. These particles can be synthesized inside of the cells and help to improve the electron transfer through the cell membrane, increasing the photocurrent by ~33 times (Blankenship et al., 2014; Zhao et al., 2015; Mouhib et al., 2019; Kim et al., 2020). An additional nano-based approach is to link or attach the cyanobacterial cells directly to the anode by using carbon nanotubes or polymers that are capable of direct charge transfer, instead of using an electron mediator (Herrero-Medina et al., 2022). Rather than using electron mediators to enhance the EET, it is also possible to artificially internalize electrons to the photosynthetic pathway. This was successfully done by application of a negative potential bias of −0.249 V (vs. SHE) that can reduce exogenous duroquinone (DQ) into duroquinol (DQH_2_) that can enter the cells and donate electrons to the plastoquinone pool in the photosynthetic pathway (Lewis et al., 2022). Although the addition of exogenous electron mediators enhances the photocurrent, it may also increase the cost of the BEPC operation. Therefore, future developments of these BPECs from laboratory scaled to usable applicative technologies will have to consider whether the addition of the chosen mediator is cost-effective in respect to its price versus the enhancement in electricity, as well as its ecological impact. The photosynthetic and respiration electron transfer tracks in cyanobacteria and the use of internal and external mediators are schematically summarized in Figure 1.

## Different configurations of cyanobacterial-based BPECs

**Figure 2:**
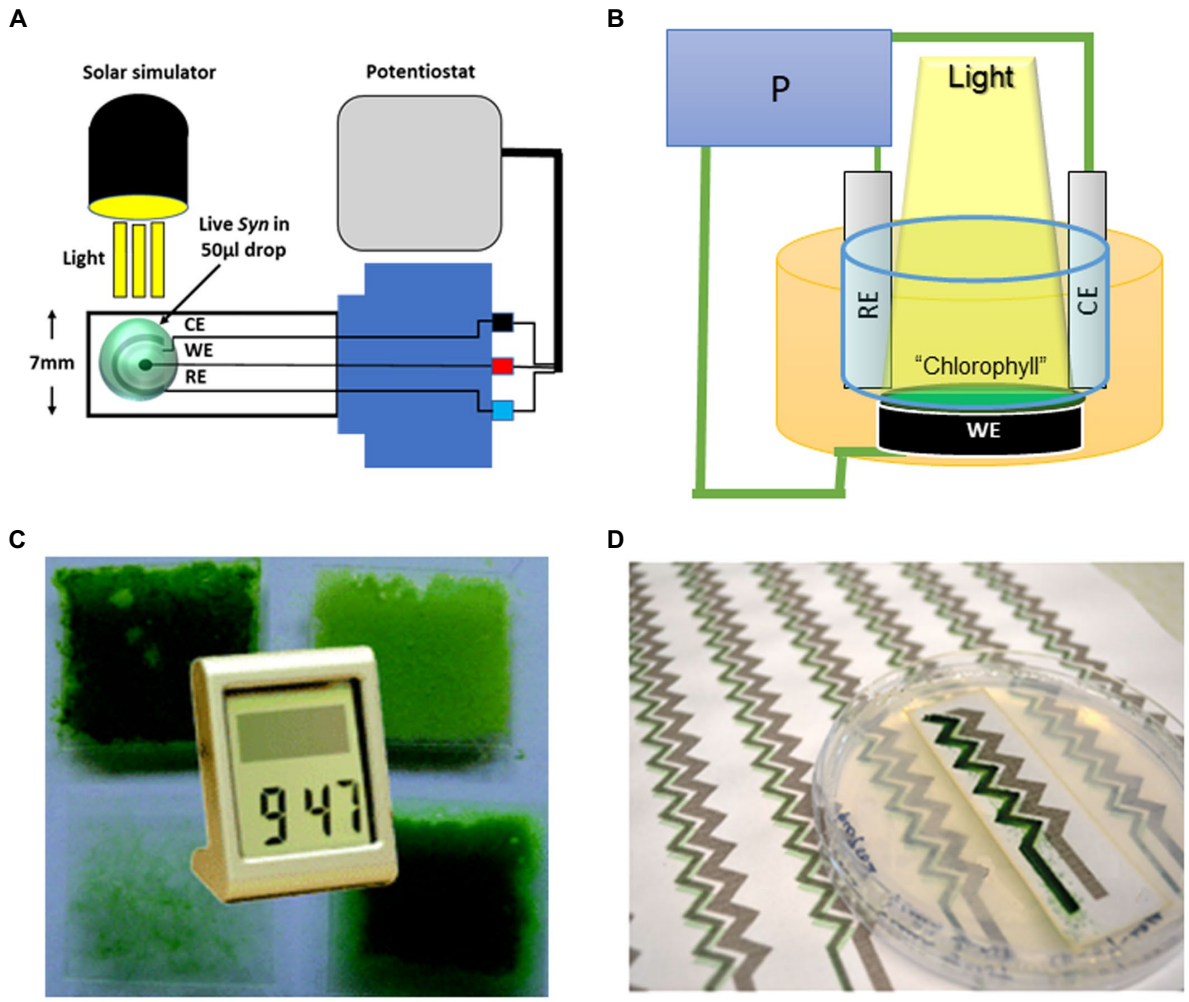
Different configurations of cyanobacterial-based BPECs. **(A)** The micro-sized BPEC is an assembly of microelectrodes in which the micro-organism, thylakoids, or isolated PS are layered in a drop of 50 μl on the circled electrodes (green circle spot). The micro-sized BPEC is used in experiments of small-scaled quantities. For example, to identify the internal mediator. Light is provided from above and care is taken that the sample in the drop would not be heated or dried during the experiment. Reprinted with permission from Shlosberg et al. (2021a). **(B)** The general and commonly used medium-sized BPEC. This commonly used BPEC contains about 50–250 ml solution in which the photosynthetic thylakoids, microorganisms, or purified PSII or PSI, indicated in the figure as “Chlorophyll,” are layered on the anode (working electrode. WE). The cathode (CE) and the reference electrode (RE) are inserted into the solution and the three are connected to the potentiostat (P). Light is provided from the above, Reprinted with permission from Saper et al. (2018). **(C)** Biofilm-based BPEC in which cyanobacteria were layered in a biofilm architecture on the anode enabling the generation of enough electricity to operate a digital watch. Reprinted with permission from Sawa et al. (2017). **(D)** Digital printing of cyanobacteria on a paper using regular ink printer in the construction of a special BPEC. Reprinted with permission from McCormick et al. (2011).

In conventional electrochemical systems that do not consist of biological components, the association of the electrodes and the reactive chemical components is arranged simply by dipping the electrodes in homogenous solutions of the reactants. The construction of living organisms’ BPECs and the designs of the association of the electrodes with the living organisms is more challenging. Most cyanobacterial species can be easily suspended in a growth medium and interact with the electrodes by layering the cells on them, making tight and close contact. For this reason, the design of a BPEC_S_ usually consists of a horizontal working electrode at the bottom, while the cells are gently placed on it (Figure 2). In case of a long-time operation of hours and days, the cell suspension should better be constantly stirred, to enable aeration and homogenous nutrient supply. Since it is not an ordinary design for an electrochemical cell, it is usually manufactured by local workshops. Another setup that has better analytic performance is the screen-printed electrodes (SPEs). One of the advantages of using SPEs is the ability to perform electrochemical measurements using small volumes of ~50 μl (Figure 2; Shlosberg et al., 2021a). This small volume allows to conduct the measurement in a homogenous suspension. The ability to use an electrochemical setup that is commercially manufactured improves the integrity of the measurements as the electrodes and their orientation are manufactured more precisely. Also, SPEs are disposable and therefore can be frequently replaced between measurements to prevent fouling effects. Moreover, a large variety of electrodes and coating materials is commercially available for SPEs.

Cyanobacterial species can form a biofilm structure (McCormick et al., 2011; Saper et al., 2018; Figures 2B,D). The architecture of the cells in a biofilm is compact and denser than in a suspension. Therefore, layering the cells in biofilm mode on the anode enables one to increase the number of cells that are in close association with the anode of the BPEC. Indeed, biofilms can produce a higher photocurrent than cyanobacterial suspensions. Another interesting technology is the utilization of digital printing of cyanobacteria. In this method, cyanobacterial suspensions are being used as ink in a standard office printer (Sawa et al., 2017). The utilization of a printer allows the making of thin cyanobacterial films on conductive surfaces whose shape can be easily designed by a standard computer software. Also, it enables easy and precise control of the design of the architecture of the BPEC (Figure 2C; Sawa et al., 2017).

## Seaweeds-based BPECs generate a significant amount of electricity

**Figure 3:**
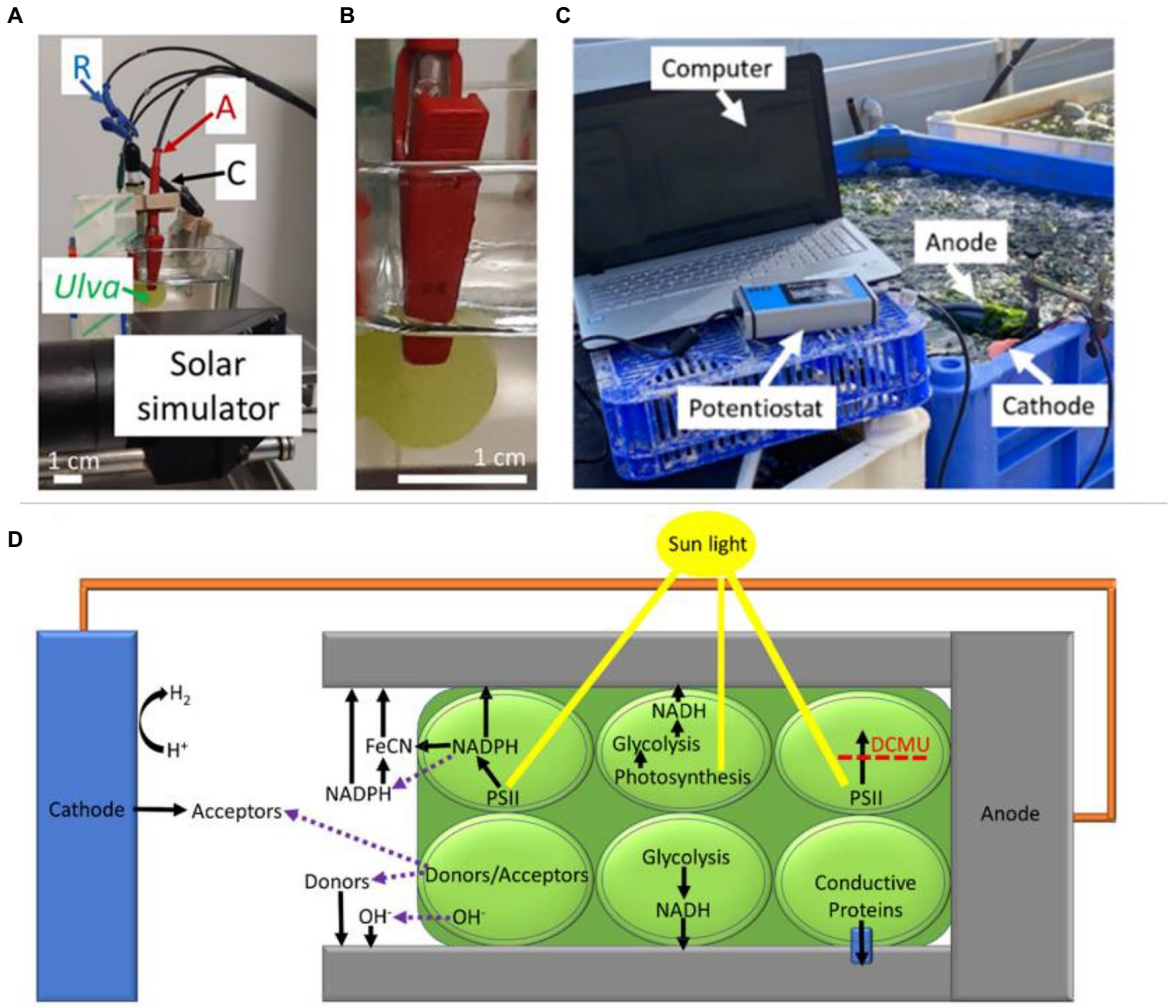
The analytical and large-scale BPECs used to obtain photocurrent from seaweeds. Reprinted with permission from Shlosberg et al. (2022a). **(A)** The analytical BPEC. A cutting of a seaweed such as *Ulva* in this picture, was electrically connected by a red clip with non-damaging flat surface serving as the anode (A). The cathode (C), and Reference electrodes (R) were placed in the solution of a synthetic seawater. A green arrow point at the section of *Ulva*. A solar simulator label shows the head of the solar simulator which illuminates the sample. **(B)** An enlargement of panel A which focuses on the connection between the anode and the *Ulva*. **(C)** A large scale BPEC. Bias-free current production of *Ulva* in its cultivation pool. The system is composed of an anode composed of a round aluminum plate and a cathode composed of a platinum wire which are dipped inside a cultivation pool with seawater and *Ulva*. The anode is held by a clamp and the cathode is placed in a sponge that is floating on the water surface. The pool is located on the seashore and contains a pipeline system that continuously streams water inside and outside of the pool. An average sunlight intensity of ~200 μmol photons m-2 s-1 was measured at the pool surface. Pieces of *Ulva* are drifting in the water stream, hitting the anode, and producing electrical current. White arrows label the components of the system including the computer that operates the potentiostat, the potentiostat, the anode, and the cathode. **(D)** Possible electron transport mechanisms in seaweeds-BPEC. Based on our findings and together with previous models which were reported for BPECs based on microalgae, cyanobacteria, non-photosynthetic bacteria, and thylakoid membranes, we propose a model for various possible EET mechanisms for the macroalgae based BPEC. The *Ulva* thallus is marked in dark green, and its cells are marked with round light green shapes. The sunlight is marked in yellow. The anode clip is marked in gray and the Pt cathode is in a blue rectangular shape. A connective spring between the anode and cathode is marked in orange. The upper three cells of the *Ulva* describe EET mechanisms that are light-dependent. The lower three cells of the Ulva describe EET mechanisms that are light-independent. A small blue cone cylinder which is located in the lower right cell indicates a hypothetical membrane-bound conductive complex. Labels indicate the different materials. Black arrows indicate the direction of potential electron transport. The purple dashed arrows indicate molecular secretion from the inner part of the *Ulva* cells to the external medium. A dashed red line that crosses a black arrow indicates the inhibition of the electron transport by DCMU.

For several decades most of the organisms based BPECs were limited to microorganisms. Recently, a BPEC using seaweeds was used for direct electricity generation (Figure 3; Shlosberg et al., 2022a). Intact seaweeds produced high photocurrent densities of up to ~50 mA/cm^2^ of which about half of the produced current was light-induced (Shlosberg et al., 2022a). This current density is about three orders of magnitudes larger than the current produced by cyanobacteria and is formed without the addition of an exogenous electron mediator. One of the factors that significantly contributes to the current generation is the tolerance of the seaweeds to the salinity of the aquatic marine environment, which can be applied as the electrolyte in the BPEC. The addition of the PSII herbicide, DCMU, eliminates the light dependent current (but not the dark current) indicating that photosynthetic electron flow through PSII is essential for the light-induced current. Among the seaweeds that were analyzed so far, the green seaweed *Ulva* produced the highest current. This can be explained by the high photosynthetic rate of this seaweed chloroplasts (Shlosberg et al., 2022a).

Unlike photosynthetic microorganisms, seaweeds are large multicellular systems with various leaf-like geometries (typically called a *thallus*) and do not form a suspension. Therefore, the configuration of standard electrochemical systems in which the electrodes are dipped into the solution is not compatible with such measurements. To address this challenge, three different configurations were designed (Shlosberg et al., 2022a). The first and a simple one is to place the seaweeds on top of a horizontal electrode and under a glass layer that prevents its flotation but enables light to penetrate and activate photosynthesis. The disadvantage of this configuration derives from the oxygen bubbles formed during photosynthesis that become a barrier between the seaweed and the electrode’s surface. A second and better configuration can be achieved by using an anode with a clip-type geometry (Figures 3A,B). The strong grasp of the clips maintains a solid attachment between the anode and the seaweed. Although such a system is good for analytical measurements, it may be less efficient for applicative uses since the seaweed may lose its viability over time and the clips block a large fraction of the light. The third and applicative configuration is in which the electrodes are dipped directly into a cultivation tank of the seaweed *Ulva*. The seaweeds move in the water stream and constantly collide with the electrodes (Figure 3C). Similar to photosynthetic microorganisms, the major electron mediator that is secreted by the seaweeds to the growth medium (sea water) is NADPH (Shlosberg et al., 2022a). In addition, it is possible to enhance the photocurrent by adding the exogenous mediator Fe(CN)_6_. In addition to the light-induced and DCMU inhibited current, a significant electric current is also obtained in the dark. The dark current could be explained by a secretion of NADH that is produced by metabolic pathways. Based on the fact that seaweeds constantly secrete OH^−^ ions in order to regulate the pH at their surface, it was suggested that these ions maybe the reducing power that produces current in dark (Shlosberg et al., 2022a).

## Electricity can be harvested from terrestrial plants of different habitat environments

**Figure 4:**
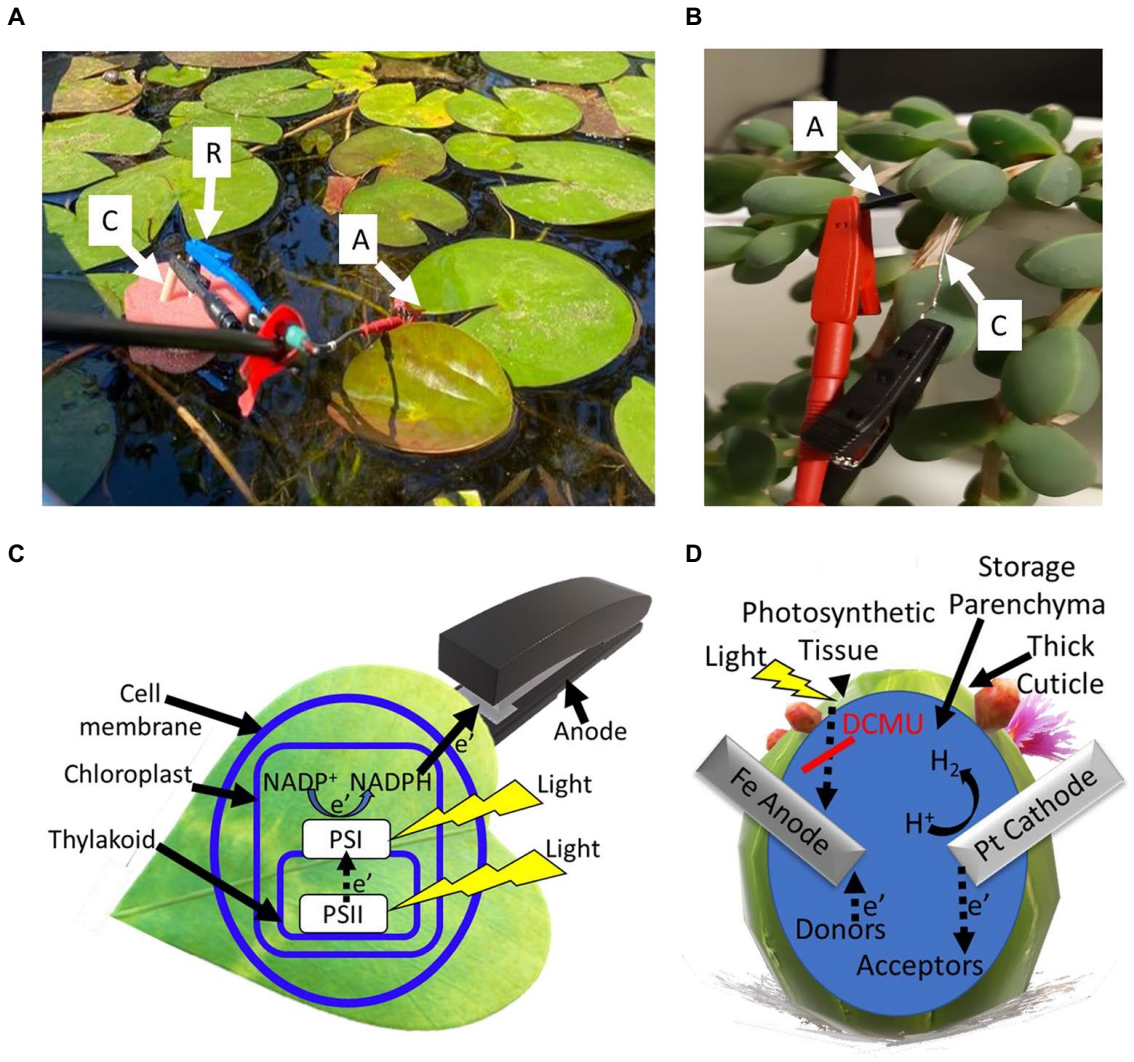
Bioelectricity production from smooth and succulent leaves. Photocurrent can be harvested from the external side of flat leaves by attaching the anode to the surface, or by insertion into the internal matrix of succulent leaves. **(A)** A picture showing a system for photocurrent production from waterlilies in their native habitat. A stainless-steel anode clip is connected directly to the external surface of the leaf (A). A platinum cathode (C) and an Ag/AgCl 3 M NaCl reference electrode (R) are dipped in the water of the pond, held by a floating sponge. **(B)** A picture showing the photocurrent production from leaves of the succulent *Corpuscularia lehmannii*. An iron anode (a) and a platinum cathode (c) are inserted into the internal matrix of the leaf. **(C)** A schematic model outlining the hypothesized electron transport pathway in the BPEC of flat leaves. Light (yellow shapes) induces photosynthesis to transfer electrons from H_2_O to PSII, PSI, and the reduction of NADP^+^ to NADPH. A portion of the NADPH molecules withdrawals from the chloroplast (light blue rectangular), transports throughout the cell membrane and wall (light blue circle) and reduces the external anode (gray shape). **(D)** A schematic model for the electron transport in the BPECs based on succulent leaves. An iron anode and a platinum cathode are inserted into the leaf. Small and enhanced electric currents are produced in dark and light, respectively. The anode is being reduced by electron donors that mediate electrons from the photosynthetic system or by redox-active molecules. The addition of DCMU (red line) eliminates the light-dependent current. The platinum cathode may reduce H^+^ ions to form H_2_ gas or other biomolecules that serve as electron acceptors. The succulent leaf has a thick cuticle that functions as a native container for the BPEC.

The recent insight that macro-organisms such as seaweeds can be utilized in BPECs has led to another class of BPECs that is based on terrestrial plants’ leaves and other green tissues (such as stems; Figure 4). Unlike cyanobacteria, microalgae, and seaweeds that are present in liquid medium, the leaves and stems of most terrestrial plants are positioned in the air. When placed in the electrolyte solution of the BPEC and under solar illumination, the leaves produce high photocurrent densities of ~10 mA/cm^2^, without the addition of an exogenous mediator (Hubenova and Mitov, 2012; Hubenova et al., 2018; Shlosberg et al., 2022c). The amount of produced electricity is largely dependent on the texture of the leaf. A softer texture of the leaf enables more trafficking of redox-active molecules between the inner part and the external anode of the BPEC. An advantage of using water plants derives from their use of native aquatic environment as the electrolyte of the BPEC (Figure 4). A different strategy is in using desert plants such as cactus and succulent species. Unlike flat leaves, whose electricity production in the BPEC derives from the release of redox-active molecules outside of the leaf and throughout the membranes and cell wall barriers, most desert plants have a thick cuticle that does not allow the trafficking of materials outside the cells. To produce electricity, it is possible to remove the external layer of the plant (Shlosberg et al., 2022c). In addition, the non-flat and thick architecture allows the insertion of bulk electrodes into the leaves (Figure 4). In this case, it was found that the plant produces electricity also in dark, which perhaps originates from the reduction of the anode by molecules that are present in the inner liquid matrix of the leaf. In fact, each leaf of a succulent plant can function as a whole independent electrochemical cell while the thick cuticle applies as a container and the inner liquid as an electrolyte (Figure 4). This may also enable increasing the voltage output by connecting several desert plant leaves in series or to increase the current by connecting several BPECs in parallel.

**Figure 5:**
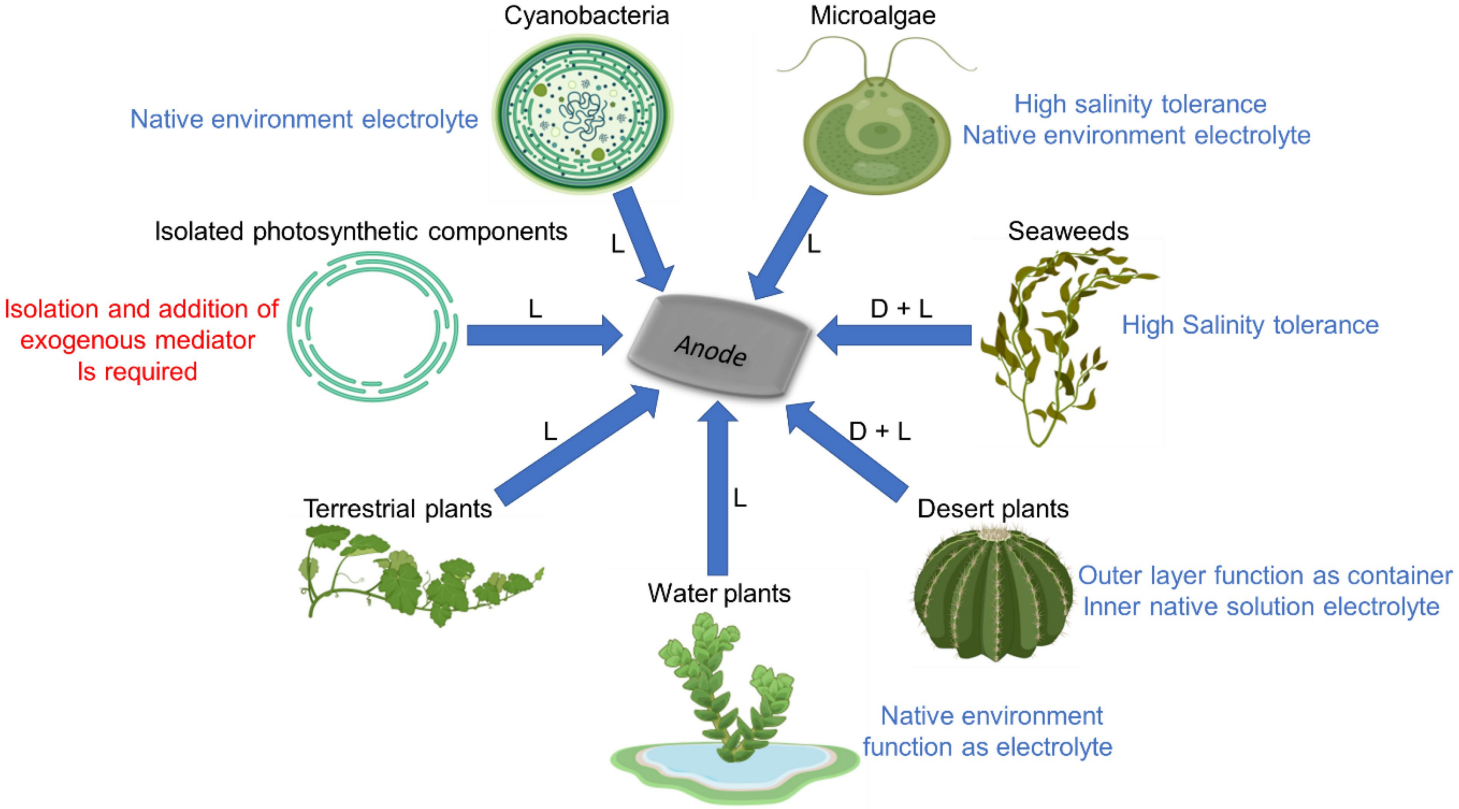
Advantages and disadvantages of using different photosynthetic organisms in BPECs. Among the different photosynthetic organisms that can produce electricity in BPECs are cyanobacteria, microalgae, seaweeds, terrestrial plants, desert plants and water plants. Electricity can be also produced from thylakoids membranes, isolated chloroplasts or purified photosystems isolated from various photosynthetic organisms. Different organisms have unique advantages for being used in BPECs. For aquatic organisms such as cyanobacteria, microalgae, seaweeds and water plants it is possible to use their environmental growth media as an electrolyte. Cyanobacteria, microalgae and all seaweed which habitatthat grow in aquatic marine environment can tolerate a high electrolyte salinity that enhance the photocurrent and therefore these are benefited for the BPEC. Desert plants have a thick cuticle that can be applied as the container and the electrochemical cell, while its inner solution can apply as the electrolyte of the electrochemical cell. Seaweeds and desert plants produce also a significant dark electricity which is enhanced in the light. Some photosynthetic organisms produce significant current using their endogenous electron mediators while an exogenous mediator must be added to thylakoids and several green algae BPECs in order to obtain significant currents. Blue arrows indicate electron flow from the organisms to the anode of the BPEC. (D, L) indicate the ability to produce a substantial electrical current in dark and light, respectively. The figure was created using BioRender.com.

A schematic illustration of the different photosynthetic organisms that were described in this review and their unique properties that can be used in BPECs is shown in Figure 5. Maximal current densities by different BPEC setups are listed in Table 1.

**Table 1:** A comparison between several BPECs.

Organism/component	Anode	Applied bias potential	Photocurrent density	Mediator/linker	Photocurrent density with mediator	References
PSI	TiO_2_	Bias free	None	polyaniline/TiO_2_	50/cm^2^	Gizzie et al., 2015
PSII	Gold	0.6 V (vs. SCE)	None	polymercapto- benzoquinone	400 nA/cm^2^	Yehezkeli et al., 2012
Thylakoids	Graphite	0.5 V (Ag/AgCI)	None	Fe(CN)_6_	100 A/cm^2^	Pinhassi et al., 2016
Chloroplasts	Carbon paper	0.3 V (vs. SCE)	None	Naphthoquinone- poly(ethylenimine) DCBQ	5 A/cm2 30 A/cm2	Hasan et al., 2017
Synehcocystis sp.6083	Graphite	0.5 V (Ag/AgCI)	5 μA/cm^2^	NADP+ NAD*	20 A/cm^2^ 20 A/cm^2^	Shlosberg et al., 2021a
*Trichodesmium Erythraeum*	Graphite	0.5 V (vsAg/AgCI)	35 μA/cm^2^	NADP+ Thiamine Cytochrome C Fe(CN)_6_	150 A/cm^2^ 140 A/cm^2^ 90 A/cm^2^ 550 A/cm^2^	Shlosberg et al., 2022b
*Dunalliela Salina*	Graphite	0.5 V (Ag/AgCI)	25 μA/cm2	NADP+ NAD+ Thiamine	130 A/cm^2^ 120 A/cm^2^ 100 A/cm^2^	Shlosberg et al., 2021b
Ulva	Stainless steel	0.5 V (Ag/AgCI)	40 mA/cm^2^	Fe(CN)_6_	80 mA/cm^2^	Shlosberg et al., 2021a
Spinach	Stainless steel	0.5 V (Ag/AgCI)	7 mA/cm^2^			Shlosberg et al., 2022c
*Nymphaeaceae*	Stainless steel	0.5 V (Ag/AgCI)	6 mA/cm^2^			Shlosberg et al., 2022c
*Corpuscularia lehmannii*	Iron	Bias free	20 μA/cm^2^			Shlosberg et al., 2022c

Example of several different BPECs that use various electrochemical setups, photosynthetic organisms or isolated photosynthetic components. The table displays the organism/isolated component, anode, applied potential bias, maximal photocurrent density (without an electron mediator), electron mediator or molecular linker that were added, and maximal photocurrent density with the mediator/linker.

## Hybrid synthetic BPECs are engineered by combining non-photosynthetic with photosynthetic organisms to symbiotically produce electricity

**Figure 6:**
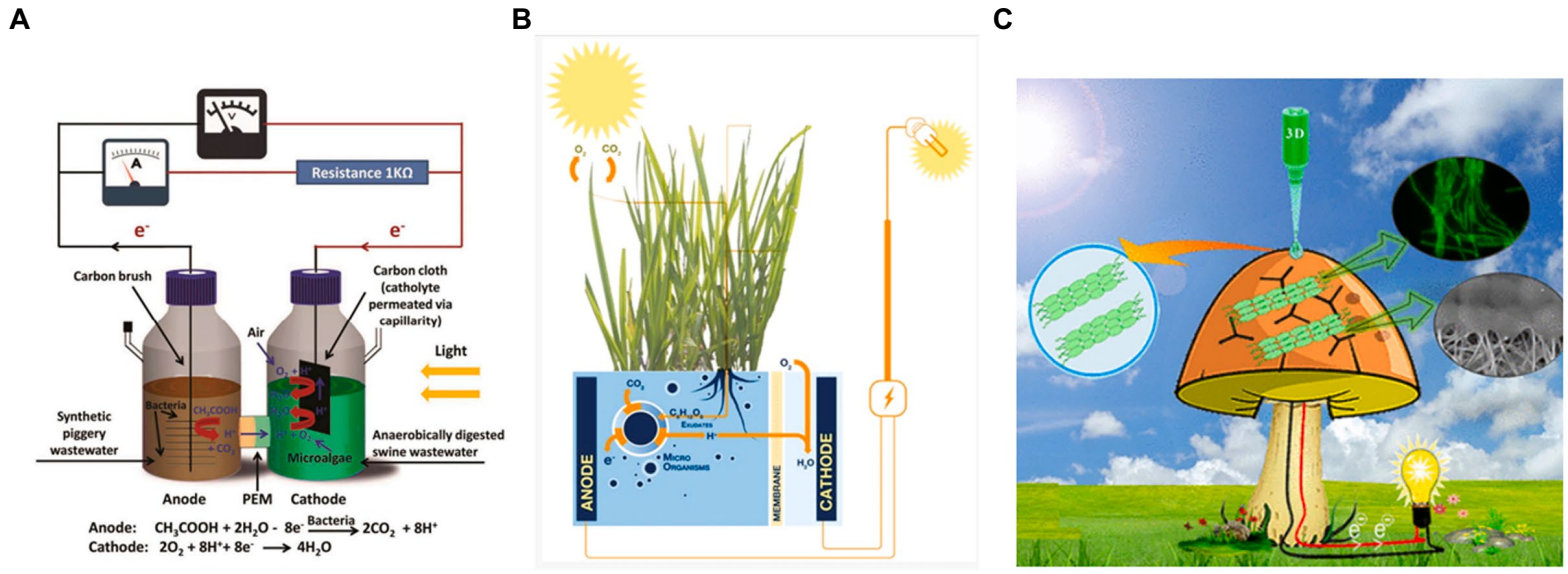
Hybrid synthetic BPECs exploit the symbiosis between different organisms. **(A)** A H-type BPEC that combines microalgae and microbial fuel cells (MFCs) from wastewaters. The MFC produces acetic acid that reduces the anode forming H^+^ ions. The H^+^ ions cross the PEM and together with the O_2_ produced by microalgal photosynthesis, oxidize the anode to form water. Reprinted with permission from Ling et al. (2019). **(B)** Plants-MFC hybrid system. Leaves conduct photosynthesis to produce glucose. The glucose is released into the soil by the roots and feed bacteria that reduce the anode placed in the soil. Reprinted with permission from Strik et al. (2008). **(C)** Cyanobacterial colonies are 3D-printed on a mushroom with graphene nanoribbons. The mushroom applies as a biocompatible substrate for cyanobacterial growth. Reprinted with permission from Joshi et al. (2018).

A novel approach to improve the performance of the BPEC has been suggested that is based on integrating several organisms in an electric production consortium. Using an H-shaped configuration, consisting of synthetic wastewater with bacteria and swine wastewater with microalgae as the anodic and cathodic half-cells, respectively. A proton exchange membrane (PEM) is placed between the half-cells (Figure 6A; Ling et al., 2019). The bacterial cells produce acetic acid that can be oxidized at the anode to form CO_2_ and H^+^. The microalgae are illuminated to produce O_2_ by photosynthetic activity that together with the H^+^ are reduced by the cathode to form H_2_O.

Another method is to integrate plants with microbial fuel cells (MFCs; Strik et al., 2008). In this method, an MFC is placed in the soil below the roots of plants. The plants that conduct photosynthesis produce sugar molecules which are being released into the soil. This sugar source can feed soil bacteria that produce electricity in an MFC. This symbiosis between the plants and the bacteria in the soil enables to preserve the bacterial viability, and in this way, prolongs the current production by the MFC (Figure 6B).

Using a third technology, a bionic mushroom in which 3D-printed colonies of cyanobacterial cells and graphene nanoribbons has being created (Joshi et al., 2018). In this configuration, the cyanobacteria have produced a photocurrent that is 8-fold higher than isotropically drop-casted cyanobacteria with the same cell density (Figure 6C).

## Concluding remarks

The research into harnessing photosynthesis to produce clean energy is attracting attention and interest because of the future possibility of being able to develop laboratory-scale BPECs into applicative and usable green energy-producing apparatuses. The photosynthetic electrons could be obtained from various and different kinds of thylakoids, cyanobacteria, different algae or plants, as described in this review. The design of the BPEC architecture, the light source, and the electrodes are the other most important constraints to be considered and designed to obtain an efficient, long-lasting system that produces significant current density on the anodes. This electric current can be utilized directly or applied to produce molecular hydrogen. Such a system must integrate an energy storage and management arrangement that will collect and store the electricity and control the output of the BEPC. Although such an applicative and practically usable system is still not at hand, the rapid development of scientific research in recent years suggests that applicative systems will be at hand sooner than is anticipated today. Introducing green energy-producing systems that are based on harnessing the photosynthesis in plants or algae would be a groundbreaking step for a cleaner world.

## Author contributions

YS, GS, and NA wrote the paper. All authors contributed to the article and approved the submitted version.

## Conflict of interest

The authors declare that the research was conducted in the absence of any commercial or financial relationships that could be construed as a potential conflict of interest.

## Publisher’s note

All claims expressed in this article are solely those of the authors and do not necessarily represent those of their affiliated organizations, or those of the publisher, the editors and the reviewers. Any product that may be evaluated in this article, or claim that may be made by its manufacturer, is not guaranteed or endorsed by the publisher.
